# Clinical and Epidemiological Profile of Multidrug-Resistant Klebsiella pneumoniae Infections in Patients With Type 2 Diabetes Mellitus

**DOI:** 10.7759/cureus.101724

**Published:** 2026-01-17

**Authors:** Adarsh Menon, Sandhya Bhat, Nayyar Iqbal, Ravichandran Kandasamy

**Affiliations:** 1 Department of General Medicine, Pondicherry Institute of Medical Sciences, Pondicherry, IND; 2 Department of Microbiology, Pondicherry Institute of Medical Sciences, Pondicherry, IND; 3 Department of Biostatistics, Pondicherry Institute of Medical Sciences, Pondicherry, IND

**Keywords:** antimicrobial resistance, carbapenem resistance, mdr klebsiella pneumoniae, polymyxin therapy, type 2 diabetes

## Abstract

The global convergence of antimicrobial resistance and type 2 diabetes mellitus (T2DM) presents significant clinical challenges. This retrospective cross-sectional study included 60 adult inpatients with T2DM and culture-confirmed multidrug-resistant (MDR) *Klebsiella pneumoniae* infections admitted between January 2022 and December 2024 at a tertiary care center in South India. The cohort had a mean age of 60.7 years, with a male predominance of 66.7% (40/60). Poor glycemic control was common (mean HbA1c 8.98%), and 51.7% (31/60) had long-standing diabetes (>10 years). Sepsis was the most frequent clinical presentation in 33.3% (20/60), followed by skin and soft tissue infections in 30.0% (18/60).

The isolates demonstrated high resistance to third-generation cephalosporins in 96.7% (58/60) and fluoroquinolones in 93.3% (56/60), while 45.0% (27/60) were carbapenem resistant. Polymyxin susceptibility was largely retained (98.3%, 59/60). The mean duration of hospital stay was 15.8 days. In-hospital mortality was low at 3.3% (2/60); however, microbiological resolution at discharge was documented in only 25.0% (15/60) of cases. These findings underscore the substantial morbidity associated with MDR *Klebsiella pneumoniae* infections in patients with T2DM despite low in-hospital mortality, highlighting the need for strengthened antimicrobial stewardship and optimized glycemic control strategies in this high-risk population. The objective of this study is to determine the clinical and demographic profiles of patients with multidrug-resistant *Klebsiella pneumoniae* infections among those with type 2 diabetes mellitus, as well as to describe antimicrobial resistance patterns and in-hospital clinical outcomes in this patient population.

## Introduction

*Klebsiella pneumoniae* is a Gram-negative, encapsulated, opportunistic pathogen that can cause severe infections, including urinary tract infections (UTIs), bloodstream infections (BSIs), respiratory tract infections, and surgical site infections [1,2]. Multidrug-resistant (MDR) strains have emerged as a global public health concern, associated with increased morbidity, mortality, and healthcare costs [3].

Patients with type 2 diabetes mellitus (T2DM) are particularly vulnerable due to chronic hyperglycemia, impaired neutrophil function, altered cytokine responses, and oxidative stress [4,5]. Prolonged hospitalization and repeated broad-spectrum antibiotic exposure contribute to the emergence and spread of multidrug-resistant organisms [6]. The convergence of carbapenem resistance and chronic hyperglycemia creates a synergistically high-risk environment that significantly narrows the window for successful empirical intervention. In regions where both diabetes and antimicrobial resistance are endemic, this clinical intersection presents a critical threat to hospital-based infection control and patient safety.

While the individual impacts of diabetes and antibiotic resistance are documented, data regarding their clinical intersection in South India remain limited. This study aimed to determine the clinical and demographic profiles, evaluate antimicrobial resistance patterns (specifically carbapenem resistance), and assess in-hospital outcomes for T2DM patients with culture-confirmed MDR *Klebsiella pneumoniae* infections.

## Materials and methods

Study design

This retrospective cross-sectional study was conducted at the Pondicherry Institute of Medical Sciences (PIMS), Pondicherry, India, after obtaining a waiver of consent from the Pondicherry Institute of Medical Sciences (PIMS) Institute Ethics Committee (IEC no: RC/2025/02).

Inclusion and exclusion criteria

Adult inpatients (≥18 years) diagnosed with T2DM and culture-confirmed MDR *Klebsiella pneumoniae* infections admitted between January 2022 and December 2024 were included. Patients with incomplete records or immunosuppression unrelated to T2DM (e.g., HIV/AIDS, chemotherapy) were excluded. The sample size was determined by taking a census of the total number of eligible patients meeting the inclusion criteria during the study period (January 2022-December 2024).

Operational definitions

Antimicrobial susceptibility testing was performed according to the Clinical and Laboratory Standards Institute (CLSI) 2022 guidelines using the Kirby-Bauer disc diffusion method [7]. Antibiotics tested included cephalosporins, fluoroquinolones, penicillins (piperacillin-tazobactam), aminoglycosides, carbapenems, sulfonamides, and polymyxins. To ensure microbiological accuracy and differentiate true infection from contamination, isolates from non-sterile sites (such as wound swabs) were included only if significant growth was observed alongside correlating clinical symptoms and elevated systemic inflammatory markers.

MDR* *was defined as resistance to at least one agent in three or more antimicrobial classes. Polymyxin susceptibility* *was confirmed via microbroth dilution to determine the minimum inhibitory concentration (MIC). Unresolved infection was defined as the absence of a documented negative culture at the time of discharge. This category included patients who were discharged with a persistently positive culture or those for whom a repeat culture was not performed prior to discharge. ICU admission was determined by institutional protocols based on the clinical need for organ support, such as mechanical ventilation or hemodynamic instability requiring vasopressor therapy.

The logistical and financial barriers to performing repeat cultures included the significant out-of-pocket cost of microbiological testing for patients, the prioritization of hospital bed turnover for clinically stable individuals, and the lack of a standardized institutional requirement for documented microbiological clearance prior to the discharge of non-critically ill patients.

Statistical analysis

Data entry was done using Microsoft Excel (Redmond, WA: Microsoft Corp.). Data analysis was done using SPSS software (Armonk, NY: IBM Corp.). Descriptive statistics, such as frequencies, percentages, and mean±standard deviation, were used for analysis. The distribution of microorganisms and their antibiotic susceptibility patterns was expressed as percentages. Categorical variables are expressed as percentages of susceptibility to each antimicrobial agent tested.

## Results

Demographic and baseline clinical characteristics

A total of 60 patients who met the inclusion criteria were included in the final analysis. The participants were predominantly composed of older males. The baseline demographic and clinical characteristics are presented in Table 1. The mean age of the patients was 60.72±10.59 years, and 40 patients (66.7%) were male. Comorbid conditions were nearly universal. Hypertension was the most common comorbidity, present in 66.7% of patients, followed closely by underlying renal conditions (55.0%) and cardiovascular disease (36.7%). Hypothyroidism (3; 5.0%), decompensated chronic liver disease (DCLD), and benign prostatic hyperplasia (BPH) were also observed in a few patients. A significant proportion of the cohort had recent contact with the healthcare system, as evidenced by a history of hospitalization within the last year (53.3%) and antibiotic use prior to the index admission (38.3%).

**Table 1:** Baseline demographics, clinical characteristics, and risk factors (n=60). T2DM: type 2 diabetes mellitus

Characteristic	Values
Demographics	
Age (years), mean±SD	60.72±10.59
Gender, n (%)	
Male	40 (66.7)
Female	20 (33.3)
Diabetes profile	
Duration of T2DM, n (%)	
≤10 years	29 (48.3)
>10 years	31 (51.7)
Glycemic control (n=43)	
Mean HbA1c (%), mean±SD	8.98±2.56
HbA1c ≥9.0%, n (%)	23 (53.5)
Diabetic complications, n (%)	17 (28.3)
Comorbidities, n (%)	
Hypertension	40 (66.7)
Underlying renal conditions	33 (55.0)
Cardiovascular disease	22 (36.7)
Risk factors, n (%)	
Prior hospitalization (within 1 year)	32 (53.3)
Prior antibiotic use (index admission)	23 (38.3)

Glycemic control and diabetic complications

The participants demonstrated markedly poor glycemic control, indicating a state of chronic hyperglycemia. The mean HbA1c was 8.98±2.56%. Among the 43 patients for whom HbA1c data were available, over half (n=23, 53.5%) had ≥9.0%, signifying severely uncontrolled diabetes. This long-standing metabolic dysregulation was further evidenced by the high prevalence of end-organ damage. Documented diabetic complications were present in 28.3% of patients, including neuropathy (13.3%), retinopathy (3.3%), and nephropathy (3.3%), with 8.3% having more than one complication.

Clinical presentation and source of infection

The clinical manifestations of MDR *K. pneumoniae* infection were severe. The most frequent clinical diagnoses upon presentation were sepsis (n=20, 33.3%) and skin and soft tissue infections (SSTI) (n=18, 30.0%). Other presentations included urinary tract infections and respiratory infections.

**Figure 1:**
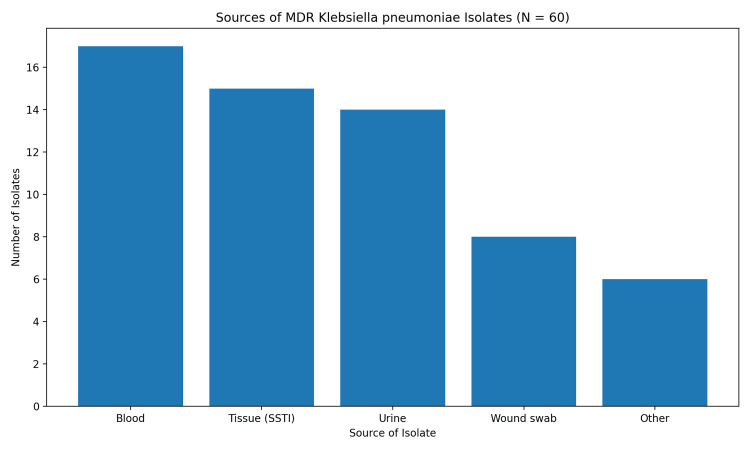
Distribution of infection sources for MDR K. pneumoniae isolates (n=60). MDR: multidrug-resistant

The primary sources from which the MDR *K. pneumoniae* isolates were cultured are shown in Figure 1. The most common source was blood (n=17, 28.3%), indicative of bacteremia, followed by tissue samples (n=15, 25.0%) from SSTIs, and urine (n=14, 23.3%). The remaining 23.4% of isolates were obtained from diverse clinical sites categorized as "others," which included wound swabs (n=8), aspirated fluids from deep-seated infections (n=4), endotracheal aspirates (n=1), and a catheter tip (n=1). While the infections were severe enough to require ICU-level care in 18.3% (n=11) of cases, the majority of patients (n=49, 81.7%) were managed in general medical wards.

Microbiological profile and antibiotic resistance patterns

A notable finding was that 30.0% of the infections were polymicrobial. In these cases, the most frequently isolated copathogens were *Pseudomonas aeruginosa* (n=6), Enterococcus species (n=5; *E. faecium *and *E. faecalis*), and *Escherichia coli* (n=3). Other isolated organisms included *Acinetobacter baumannii*, Enterobacter species, and *Streptococcus agalactiae*. The presence of these diverse copathogens, particularly in skin and soft tissue infections (SSTIs), underscores the therapeutic challenge in this diabetic cohort. The antimicrobial susceptibility testing revealed a profile of extensive resistance. As detailed in Table 2, resistance to commonly used broad-spectrum antibiotics was nearly universal. An overwhelming 96.7% of isolates were resistant to third-generation cephalosporins, and 93.3% were resistant to fluoroquinolones, rendering these entire classes of antibiotics empirically useless for this patient population.

**Table 2:** Antibiotic resistance patterns of MDR Klebsiella pneumoniae isolates (n=60). MDR: multidrug-resistant

Antibiotic class	Number of resistant isolates	Percentage
Cephalosporins	58	96.7
Fluoroquinolones	56	93.3
Penicillins	50	83.3
Aminoglycosides	34	56.7
Carbapenems	27	45.0
Sulfonamides	20	33.3
Nitrofurans	11	18.3
Tetracyclines	4	6.7
Polymyxins	1	1.7

The most clinically significant finding was the exceptionally high rate of carbapenem resistance. A total of 27 isolates (45.0%) were classified as carbapenem-resistant *Klebsiella pneumoniae* (CRKP). However, susceptibility was largely preserved to last-line agents, with only 1 isolate (1.7%) demonstrating resistance to polymyxins.

Laboratory markers

Laboratory findings at admission reflected a state of significant systemic inflammation and physiological stress, as summarized in Table 3. The mean total leukocyte count (TLC) was elevated at 14,095 cells/mm³, and the mean CRP was 98.28 mg/L. Evidence of renal impairment was widespread, with a mean serum creatinine of 1.89 mg/dL. Anemia was also common, with a mean hemoglobin level of 9.87 g/dL.

**Table 3:** Key laboratory findings at admission (n=60).

Laboratory characteristic	Number	Percentage
HbA1c (mmol/mol) (n=43)		
<7.0	14	32.6
7.0-8.9	6	14.0
≥9.0	23	53.5
Total leukocyte count (cells/mm³) (n=60)		
<11 (normal)	19	31.7
11-15 (mild elevation)	16	26.7
>15 (high elevation)	25	41.7
Serum creatinine (mg/dL) (n=58)		
Normal (≤1.2)	28	48.3
Mildly elevated (1.3-2.0)	9	15.5
Severely elevated (>2.0)	21	36.2
C-reactive protein (CRP) (mg/L) (n=43)		
<10 (normal)	3	7.0
10-100 (mild to moderate elevation)	21	48.8
>100 (high elevation)	19	44.2
Random glucose (mg/dL) (n=50)		
<140 (normal)	5	10.0
140-199 (impaired)	7	14.0
≥200 (diabetes-level)	38	76.0

Clinical outcomes

The in-hospital mortality rate for the cohort was relatively low at 3.3% (n=2). However, the burden of morbidity was immense. The mean duration of hospital stay was prolonged at 15.78±10.14 days, with 43.3% of patients requiring hospitalization for more than two weeks (Table 4). Most concerning was the low rate of documented infection resolution at the time of discharge. A resolution was noted in only 25.0% (n=15) of cases. This indicates that while most patients survived the acute infectious episode, a substantial majority (75%) were discharged with persistent or unresolved infections, posing a significant risk for relapse and further community dissemination of these resistant pathogens.

**Table 4:** Clinical outcomes of participants (n=60).

Outcomes	Number	Percentage
Duration of hospital stay (days)		
≤7 days	13	21.7
8-14 days	21	35.0
>14 days	26	43.3
Mortality		
Yes	2	3.3
No	58	96.7

Therapeutic management

An analysis of the therapeutic regimens administered demonstrates substantial discordance between empirical antibiotic use and in vitro susceptibility patterns (Table 5). Penicillins (41.7%) and cephalosporins (21.7%) were frequently used despite high resistance rates of 83.3% and 96.7%, respectively. Carbapenems were administered in 25.0% of patients, even though 45.0% of isolates were carbapenem-resistant. Polymyxins were used in 16.7% of cases, corresponding to preserved susceptibility in 98.3% of isolates.

**Table 5:** Antibiotic classes administered for treatment of MDR Klebsiella pneumoniae infections (n=60). MDR: multidrug-resistant

Antibiotic class	Number of patients treated	Usage rate (%)
Penicillins	25	41.7
Carbapenems	15	25.0
Cephalosporins	13	21.7
Aminoglycosides	11	18.3
Tetracyclines	11	18.3
Polymyxins	10	16.7
Lincosamides	4	6.7
Oxazolidinones	4	6.7
Nitroimidazoles	3	5.0
Fluoroquinolones	2	3.3
Nitrofurans	2	3.3
Glycopeptides	1	1.7
Macrolides	1	1.7
Antifungals	1	1.7
Sulfonamides	1	1.7

## Discussion

In this study of 60 patients with type 2 diabetes mellitus (T2DM) and multidrug-resistant (MDR) *Klebsiella pneumoniae* infection, several clinically important findings were observed. The mean age of 60.7±10.6 years and male predominance are consistent with earlier studies showing a higher burden of MDR infections among older diabetic men due to greater comorbidity and healthcare exposure [8,9].

Glycemic control and disease duration

Poor glycemic control was evident, with a mean HbA1c of 8.98±2.56%, and more than half of the patients had HbA1c ≥9.0%. Long-standing diabetes (>10 years) was present in 51.7% of patients. Similar findings were reported by Liu et al., who demonstrated that poor glycemic control (HbA1c >8%) significantly increased the risk and severity of *K. pneumoniae* infections in diabetic patients [10]. Chronic hyperglycemia is known to impair neutrophil function and reduce host immune defense, hence predisposing to MDR infections [11].

Comorbidities and prior exposures

Comorbid conditions were highly prevalent, particularly hypertension (66.7%) and renal impairment (55.0%). Previous studies have highlighted chronic kidney disease and prior antibiotic exposure as major risk factors for colonization and infection with MDR *K. pneumoniae* [12]. The presence of cardiovascular disease (36.7%) and recent hospitalization (53.3%) further reflects the high-risk nature of this cohort.

Clinical profile and infection source

Sepsis (33.3%) and skin and soft tissue infections (30.0%) were the most common clinical presentations. Blood (28.3%), tissue (25.0%), and urine (23.3%) were the predominant sites of isolation. A similar distribution was reported by Maraolo et al., who noted bloodstream and urinary tract infections as the most frequent presentations in carbapenem-resistant *Klebsiella pneumoniae* (CRKP) cohorts [13]. The need for intensive care in 18.3% of patients aligns with earlier findings that approximately 15-25% of MDR *K. pneumoniae* infections require ICU-level management [14].

Antibiotic resistance patterns

Resistance to third-generation cephalosporins (96.7%) and fluoroquinolones (93.3%) was nearly universal, and 45.0% of isolates were carbapenem-resistant. This closely parallels the resistance patterns described in large-scale Asian surveillance studies, where cephalosporin and fluoroquinolone resistance rates exceed 90%, and carbapenem resistance ranges from 40% to 50% [15]. The low polymyxin resistance (1.7%) in this study is encouraging and similar to the <5% reported in most global series [16].

Therapeutic management and its impact on clinical outcomes

Despite extensive resistance to commonly used agents, in-hospital mortality remained low, whereas morbidity was substantial, reflected by prolonged hospitalization and a high rate of unresolved infection at discharge. The early initiation of last-line agents, such as polymyxins, in a limited subset of critically ill patients may have contributed to short-term clinical stabilization, particularly in those with severe infection and preserved in vitro susceptibility. However, given the limited number of patients receiving polymyxins and the retrospective design of this study, prospective studies are required to conclusively determine the impact of polymyxin therapy on survival and microbiological outcomes in this population [12,16]. While polymyxins demonstrated high in vitro susceptibility in this cohort (98.3%), recent evidence has increasingly highlighted their limitations, including significant nephrotoxicity and inconsistent clinical efficacy compared to newer therapeutic agents [17]. Current global guidelines now favor newer beta-lactam/beta-lactamase inhibitors over polymyxin-based regimens when available. However, in resource-limited settings where access to newer antibiotics is restricted by cost or availability, polymyxins often remain the only viable last-line option for carbapenem-resistant infections, necessitating careful monitoring for toxicity.

This therapeutic mismatch provides a compelling explanation for the combination of prolonged hospitalization (mean stay 15.78 days) and the high rate of unresolved infection at discharge (75.0%). Importantly, unresolved infection in this study was defined by the absence of documented microbiological clearance at discharge and did not necessarily imply ongoing clinical instability. In several cases, patients demonstrated clinical improvement and were discharged once clinically stable, while repeat cultures were not performed due to logistical constraints, cost considerations, or perceived lack of immediate clinical indication.

Consequently, what might otherwise have resulted in early mortality may instead have evolved into persistent or incompletely documented infection, shifting the clinical and economic burden to the post-discharge setting and increasing the risk of ongoing transmission of highly resistant pathogens.

Clinical outcomes

The mean hospital stay was 15.8±10.14 days, with 43.3% hospitalized for more than two weeks. Studies from tertiary centers in China and India have similarly reported median hospital durations exceeding 14 days for MDR *K. pneumoniae* infections [18,19].

A central and unexpected finding of this study was the in-hospital mortality rate of 3.3%, which is a stark outlier when contextualized within the existing literature on carbapenem-resistant *Klebsiella pneumoniae* (CRKP) infections. This figure is substantially lower than rates reported in numerous comparable studies, which typically range from 33% to 50% [20]. For instance, a recent study from an ICU in Eastern India reported a 44% mortality rate for patients with CRKP infections, a risk 1.9 times higher than that for patients with carbapenem-sensitive strains [21]. One study investigating carbapenem-resistant Enterobacterales infections found that the 30-day hospital mortality in diabetic patients was 48.9% [22].

Limitations

First, its retrospective, single-center design may limit the generalizability of the results, as patient demographics, regional resistance patterns, and clinical practices can vary significantly. A major limitation is the lack of post-discharge follow-up. Given the alarming finding that 75.0% of patients were discharged with unresolved infections, the absence of follow-up data prevents the assessment of crucial long-term outcomes and the true overall cost of care. The lack of molecular characterization for carbapenemase types (e.g., NDM or KPC) and the non-standardized timing of follow-up cultures are additional limitations.

## Conclusions

In patients with type 2 diabetes mellitus, multidrug-resistant *Klebsiella pneumoniae* infections represent a substantial clinical challenge characterized by high antimicrobial resistance and significant morbidity. Although in-hospital mortality was low in this cohort, patients experienced prolonged hospital stays and a high rate of unresolved infection at discharge, indicating that survival often came at the cost of persistent disease burden.

These findings highlight the need for early recognition of high-risk diabetic patients, optimized glycemic control, and judicious use of effective antimicrobial therapy guided by susceptibility patterns. Strengthening antimicrobial stewardship practices and developing structured post-discharge follow-up strategies are essential to reduce ongoing morbidity and limit the community spread of resistant pathogens in this vulnerable population.

## References

[1] Podschun R, Ullmann U (1998). Klebsiella spp. as nosocomial pathogens: epidemiology, taxonomy, typing methods, and pathogenicity factors. Clin Microbiol Rev.

[2] Russo TA, Marr CM (2019). Hypervirulent Klebsiella pneumoniae. Clin Microbiol Rev.

[3] (2017). Prioritization of pathogens to guide discovery, research and development of new antibiotics for drug-resistant bacterial infections, including tuberculosis. https://www.who.int/publications/i/item/WHO-EMP-IAU-2017.12.

[4] Joshi N, Caputo GM, Weitekamp MR, Karchmer AW (1999). Infections in patients with diabetes mellitus. N Engl J Med.

[5] Casqueiro J, Casqueiro J, Alves C (2012). Infections in patients with diabetes mellitus: a review of pathogenesis. Indian J Endocrinol Metab.

[6] Magiorakos AP, Srinivasan A, Carey RB (2012). Multidrug-resistant, extensively drug-resistant and pandrug-resistant bacteria: an international expert proposal for interim standard definitions for acquired resistance. Clin Microbiol Infect.

[7] Clinical and Laboratory Standards Institute (2022). Performance standards for antimicrobial susceptibility testing, 32nd edition. Performance Standards for Antimicrobial Susceptibility Testing CLSI supplement M100.

[8] Salawudeen A, Raji YE, Jibo GG (2023). Epidemiology of multidrug-resistant Klebsiella pneumoniae infection in clinical setting in South-Eastern Asia: a systematic review and meta-analysis. Antimicrob Resist Infect Control.

[9] Chen J, Ma H, Huang X (2022). Risk factors and mortality of carbapenem-resistant Klebsiella pneumoniae bloodstream infection in a tertiary-care hospital in China: an eight-year retrospective study. Antimicrob Resist Infect Control.

[10] Liu B, Yi H, Fang J, Han L, Zhou M, Guo Y (2019). Antimicrobial resistance and risk factors for mortality of pneumonia caused by Klebsiella pneumoniae among diabetics: a retrospective study conducted in Shanghai, China. Infect Drug Resist.

[11] Peleg AY, Weerarathna T, McCarthy JS, Davis TM (2007). Common infections in diabetes: pathogenesis, management and relationship to glycaemic control. Diabetes Metab Res Rev.

[12] Tumbarello M, Trecarichi EM, De Rosa FG (2015). Infections caused by KPC-producing Klebsiella pneumoniae: differences in therapy and mortality in a multicentre study. J Antimicrob Chemother.

[13] Maraolo AE, Corcione S, Grossi A (2021). The impact of carbapenem resistance on mortality in patients with Klebsiella pneumoniae bloodstream infection: an individual patient data meta-analysis of 1952 patients. Infect Dis Ther.

[14] Gomez-Simmonds A, Uhlemann AC (2017). Clinical implications of genomic adaptation and evolution of carbapenem-resistant Klebsiella pneumoniae. J Infect Dis.

[15] Qian Y, Bi Y, Liu S, Li X, Dong S, Ju M (2021). Predictors of mortality in patients with carbapenem-resistant Klebsiella pneumoniae infection: a meta-analysis and a systematic review. Ann Palliat Med.

[16] Kumarasamy KK, Toleman MA, Walsh TR (2010). Emergence of a new antibiotic resistance mechanism in India, Pakistan, and the UK: a molecular, biological, and epidemiological study. Lancet Infect Dis.

[17] Bell TD, Park SC (2023). Colistin - that was fun, but now we're done. NEJM Evid.

[18] Falagas ME, Kasiakou SK (2005). Colistin: the revival of polymyxins for the management of multidrug-resistant Gram-negative bacterial infections. Clin Infect Dis.

[19] Wang Z, Qin RR, Huang L, Sun LY (2018). Risk factors for carbapenem-resistant Klebsiella pneumoniae infection and mortality of Klebsiella pneumoniae infection. Chin Med J (Engl).

[20] Panda S, Dash A, Chhotray P, Nayak B, Mouli TC, Mishra SB (2022). Risk factors and clinical outcomes of carbapenem-resistant Klebsiella pneumonia infection in intensive care unit: a retrospective observational study in a tertiary care hospital in Eastern India. Int J Crit Illn Inj Sci.

[21] Pattolath A, Adhikari P, Pai V (2024). Carbapenemase-producing Klebsiella pneumoniae infections in diabetic and nondiabetic hospitalized patients. Cureus.

[22] Aon M, Aoun AH, Al Shami A (2024). Association of diabetes mellitus with increased mortality in carbapenem-resistant Enterobacterales infections. Cureus.

